# Supplementary material for: Polyketide synthase gene clusters in the ericoid mycorrhizal fungus Oidiodendron maius: genome-wide prediction, expression profiling, and functional characterization of knockout mutants
Source: Front Plant Sci. 2026 Sep 16;17:1924430. doi: 10.3389/fpls.2026.1924430 (PMC13623903; doi:10.3389/fpls.2026.1924430)
Supplement: Supplementary file 1 [file SupplementaryFile1.zip › Supplementary Materials/Supplementary Folder 1.zip!/Oidma1_AssemblyScaffolds_Repeatmasked/knownclusterblast/cluster47/jgi.p_Oidma1_136009_mibig_hits.html]

| MIBiG Protein | Description | MIBiG Cluster | MiBiG Product | % ID | % Coverage | BLAST Score | E-value |
| --- | --- | --- | --- | --- | --- | --- | --- |
| AMR44278.1 | putative\_short\_chain\_dehydrogenase\_reductase | BGC0001398\_c5 | Phomopsins\_biosynthetic\_gene\_cluster | 80 | 100.342465753 | 457 | 1.3e-128 |
| ABB69757.1 | PlaU | BGC0000654 | Phenalinolactone\_biosynthetic\_gene\_cluster | 30 | 93.4931506849 | 92 | 1.1e-18 |
| ABC42552.1 | putative\_3-ketoacyl\_ACP\_reductase | BGC0000698 | Hygromycin\_A\_biosynthetic\_gene\_cluster | 27 | 92.4657534247 | 88 | 1.2e-17 |
| ALG65299.1 | Cal37 | BGC0001297 | WS9326\_biosynthetic\_gene\_cluster | 30 | 91.4383561644 | 87 | 2.7e-17 |
| AAZ78333.1 | OxyJ | BGC0000254 | Oxytetracycline\_biosynthetic\_gene\_cluster | 28 | 92.8082191781 | 80 | 3.2e-15 |
| ADB23395.1 | dehydrogenase | BGC0001062 | TLN-05220\_biosynthetic\_gene\_cluster | 28 | 93.8356164384 | 78 | 1.2e-14 |
| AAA65204.1 | daunorubicin-doxorubicin\_polyketide\_synthase | BGC0000218 | Doxorubicin\_biosynthetic\_gene\_cluster | 28 | 95.5479452055 | 77 | 3.6e-14 |
| CAM58804.1 | BenL\_protein | BGC0000204 | Benastatin\_biosynthetic\_gene\_cluster | 29 | 92.4657534247 | 76 | 4.7e-14 |
| CAI94720.1 | putative\_oxyacyl-(acyl\_carrier\_protein)\_reductase | BGC0000141 | Rubradirin\_biosynthetic\_gene\_cluster | 27 | 92.4657534247 | 76 | 8e-14 |
| AAL15583.1 | Sim5 | BGC0000270 | Simocyclinone\_biosynthetic\_gene\_cluster | 28 | 93.8356164384 | 74 | 2.3e-13 |
| AAK06787.1 | putative\_ketoreductase\_SimA6 | BGC0001072 | Simocyclinone\_biosynthetic\_gene\_cluster | 28 | 93.8356164384 | 74 | 2.3e-13 |
| WP\_030269270.1 | ketoacyl\_reductase | BGC0001308 | Simocyclinone\_D9\_/\_simocyclinone\_D10\_/\_simocyclinone\_D11 | 28 | 93.4931506849 | 73 | 4e-13 |
| CUW01207.1 | putative\_3-oxoacyl-ACP\_reductase | BGC0001359\_c2 | PM100117\_/\_PM100118\_biosynthetic\_gene\_cluster | 29 | 94.8630136986 | 73 | 4e-13 |
| CAA12013.1 | SnoaD | BGC0000250 | Nogalamycin\_biosynthetic\_gene\_cluster | 29 | 94.8630136986 | 72 | 6.8e-13 |
| CAM59605.1 | putative\_reductase | BGC0000297 | Aeruginoside\_biosynthetic\_gene\_cluster | 24 | 94.5205479452 | 71 | 1.5e-12 |
| AEE65465.1 | ketoreductase | BGC0000223 | Fluostatin\_biosynthetic\_gene\_cluster | 29 | 93.1506849315 | 71 | 2e-12 |
| AAF81727.1 | putative\_ketoreductase\_EncD | BGC0000220 | Enterocin\_biosynthetic\_gene\_cluster | 28 | 94.5205479452 | 69 | 7.5e-12 |
| CAD62198.1 | Ata4\_protein | BGC0000873 | A201A\_biosynthetic\_gene\_cluster | 26 | 92.8082191781 | 68 | 1.3e-11 |
| BAB72043.1 | AknA | BGC0000191 | Aclacinomycin\_biosynthetic\_gene\_cluster | 26 | 93.8356164384 | 67 | 3.7e-11 |
| AAF70104.1 | AknA | BGC0000192 | Aclacinomycin\_biosynthetic\_gene\_cluster | 27 | 93.8356164384 | 66 | 4.8e-11 |
| AET51861.1 | 3-ketoacyl\_ACP\_reductase | BGC0001138 | A201A\_biosynthetic\_gene\_cluster | 25 | 91.095890411 | 63 | 4.1e-10 |
| ABQ04140.1 | short-chain\_dehydrogenase/reductase\_SDR | BGC0000838 | Flexirubin\_biosynthetic\_gene\_cluster | 24 | 94.1780821918 | 57 | 2.3e-08 |
| CBK62720.1 |  | BGC0001115 | Thiomarinol\_biosynthetic\_gene\_cluster | 27 | 91.4383561644 | 54 | 1.9e-07 |
